# Social Determinants of Disease: HIV and COVID-19 Experiences

**DOI:** 10.1007/s11904-021-00595-6

**Published:** 2022-02-02

**Authors:** Raiza M. Beltran, Ian W. Holloway, Chenglin Hong, Ayako Miyashita, Luisita Cordero, Elizabeth Wu, Katherine Burris, Paula M. Frew

**Affiliations:** 1 David Geffen School of Medicine, Department of Infectious Diseases, UCLA Global HIV Prevention Research Program, 10833 Le Conte Avenue, Los Angeles, CA 90095 USA; 2 UCLA Hub for Health Intervention, Policy and Practice (HHIPP), CA Los Angeles, USA; 3 Department of Social Welfare, School of Public Affairs, UCLA Luskin, Los Angeles, CA USA; 4 California HIV/AIDS Research Program, Los Angeles, CA USA; 5 UNLV School of Public Health, UNLV Population Health & Health Equity Initiative, University of Nevada, Las Vegas, NV USA

**Keywords:** HIV/AIDS, COVID-19, Social determinants of health, Racial/ethnic minorities, Health disparities, Health equity, Structural racism

## Abstract

**Purpose of Review:**

The differential impact of the COVID-19 and HIV pandemics on marginalized communities has renewed calls for more robust and deeper investigation into structural and social causes of health inequities contributing to these infections, including underlying factors related to systematic racism. Using the Social Determinants of Health (SDOH) framework, we analyzed parallel and divergent factors associated with COVID-19 and HIV/AIDS and the prevalence of disparate disease in diverse communities. We utilized PRISMA guidelines to identify relevant literature (N = 210 articles) that resulted in a review of 125 articles included in our synthesis.

**Recent Findings:**

With racial health inequities as a core contributor to disease vulnerability, we also identified other factors such as economic stability, social and community support, the neighborhood and built environment, healthcare access and quality, and education access and quality as important socioecological considerations toward achieving health equity. Our review identifies structural and systematic factors that drive HIV and COVID-19 transmission.

**Summary:**

Our review highlights the importance of not solely focusing on biomedical interventions as solutions to ending HIV and COVID-19, but rather call for building a more just public health and social service safety net that meets the needs of people at the intersection of multiple vulnerabilities.

## Introduction

**Fig. 1:**
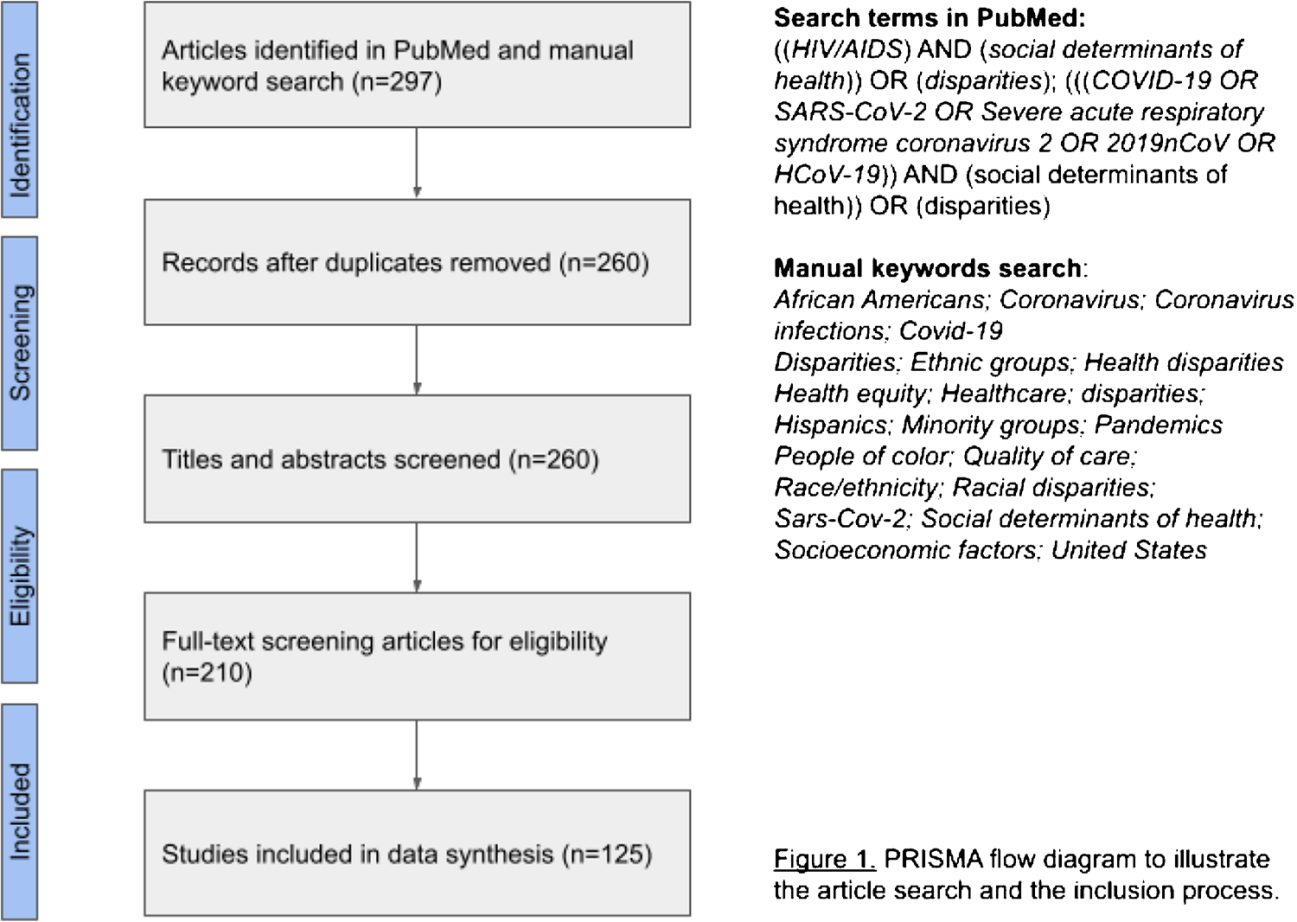
PRISMA Flow Diagram

The differential impact of the COVID-19 and HIV pandemics on marginalized communities in the USA raised serious concerns among public health practitioners [1••, 2••, 3, 4••]. New data reveal the extent of rapid transmission of COVID-19 among US residents identifying as Black/African American [5, 6••], Hispanic/Latino [7, 8], Indigenous/Native American [9•] as well as those who live in densely concentrated, low-income neighborhoods [10•, 11•], and work in essential services [12, 13]. This has renewed calls for more robust and deeper investigation to disentangle the structural and social causes of health inequities, including systematic racism [1••, 4••, 14••, 15]. In an effort to answer this call, and in following the CDC’s recent declaration of structural racism as a serious public health threat [16], we used the CDC’s Social Determinants of Health (SDOH) framework defined as “conditions in the places where people live, learn, work,” to better understand how the marginalization of certain communities can leave them vulnerable to both COVID-19 and HIV (See Fig. 1, Table 1).

**Table 1:** Conditions in the places where people live, learn, work

COVID-19	Racial determinants *Specific factors*	HIV
[4••, 5, 8, 31–36, 41]	*Black Americans: cases, hospitalizations, deaths*	
[7, 12, 19, 37, 38, 53, 60•]	*Hispanic/Latino Americans: cases, hospitalizations, deaths*	
	*Black and Hispanic/Latino gay, bisexual and other men who have sex with men (GBMSM): incidence, prevention and treatment*	[14••, 42, 46, 47, 50]
	*Black and Hispanic/Latino heterosexual women: incidence, prevention and treatment*	[43, 44, 46, 48, 49, 51]
	CDC social determinants of health *Specific factors*	HIV
	Economic stability: income inequities and employment	
[1••, 4••, 9•, 11•, 12, 15, 22, 25, 52, 53]	*Economically disadvantaged/poverty*	[1••, 4••, 15, 45, 60•, 61–64, 65•, 66–74]
[1••, 12, 13, 23, 33, 54–59]	*Employment instability & work related outcomes*	[75, 76]
	Neighborhoods and built environment: housing and population density	
[7, 10•, 11•, 12, 23, 39, 52, 58, 77, 78, 80–84]	*Housing density, displacement & instability*	[72, 77, 85–87]
[23, 58, 79, 80]	*Population and residential density*	[61]
	Social and community support: stigma, discrimination, norms and social networks	
[2••, 14••, 88, 90, 91, 92•, 93, 97]	*Stigma and discrimination*	[14••, 47, 65•, 67, 73, 88, 89, 95, 98•, 99–102]
[94–96]	*Social networks, community and institutional supports*	[47, 65•, 67, 73, 101, 105]
	Healthcare access and quality	[1••, 12, 89] [47, 111] [102] [112]
[1••, 10•, 12, 33, 54, 89, 95, 96, 106–108]	*Mistrust in healthcare institutions*	[1••, 12, 47, 89, 102, 112]
[10•, 12, 33, 53, 54, 82, 96, 107–110]	*Barriers to access: health insurance coverage, reduced funding of services and other structural factors*	[2••, 51, 67, 68, 98•, 101]
	Educational access and quality	
[23, 26, 28, 30, 53, 55]	*Education achievement*	[45, 87, 100, 112, 113]

## Search Strategy

**Fig. 2:**
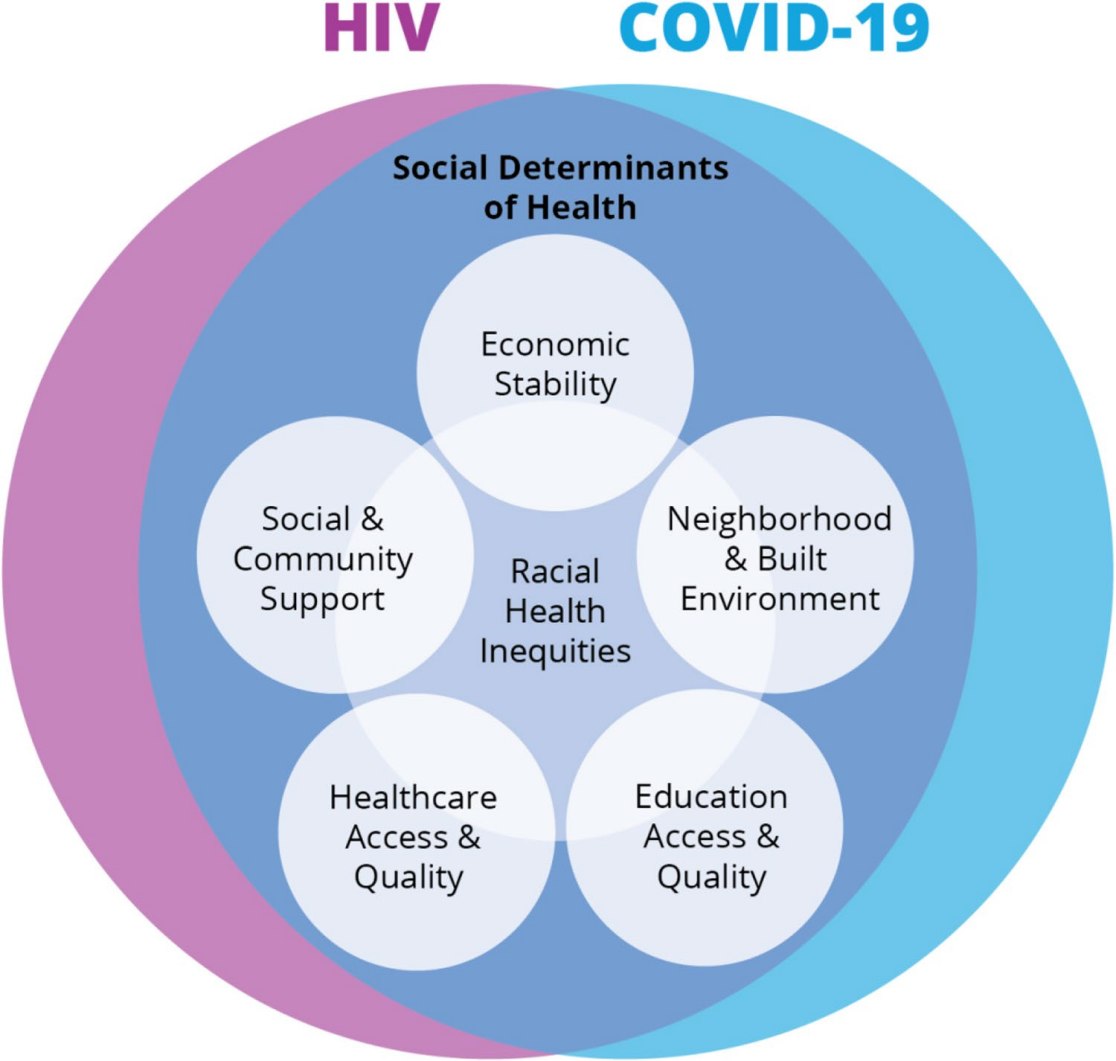
The CDC’s Social Determinants of Health (SDOH) Framework and Communities’ Vulnerability to COVID-19 and HIV

Adapting PRISMA guidelines, we conducted a literature search on PubMed for publications and used a combination of keywords, phrases, and Medical Subject Headings (MeSH) that included *HIV/AIDS*, *social determinants of health*, *disparities*, and *COVID-19* [17]. We also did a manual search for other relevant studies. We executed the initial search in October 2020 and updated the search in March 2021, which altogether yielded 286 articles. Three authors (RB, CH, LC) independently screened the titles and abstracts to narrow down eligible articles to 199 for full text review. The included articles provided empirical and original data and were published in the past 10 years and in English. Selected articles with qualitative data provided relevant context and supportive evidence. See Fig. 1 for an outline of search strategy and study selection. It is important to note that this is a limited review highlighting the features that stand out in the landscape of recent research useful for comparing COVID-19 and HIV. A broader review of published articles from multiple databases would provide a more comprehensive perspective (Fig. 2).

## COVID-19 and HIV–Related Health Inequities: Examining Race

### COVID-19 and Racial Health Inequities

Medical sociologists Phelan and Link [18] described racism as a “fundamental cause of health inequality” that can lead to persistent and differentiated health outcomes among racial and ethnic groups, independent of their socioeconomic status. Recent research findings on COVID-19 and HIV, substantiate Phelan and Link’s assertion [18]. For COVID-19–related cases [19–23], hospitalizations [7, 24–26], and deaths [27–30], communities of color had higher rates than their white counterparts. One such study assessed ten major US cities that experienced COVID-19 surges and found that counties with more poverty and a substantial non-white population had a COVID-19 incident infection rate at 8 times higher and death rate at 9 times greater than similar counties with a substantial white population [11•]. More specifically, Black Americans, who account for only 13% of the total US population, carry a greater COVID-19–related burden in positive cases [5, 31–33]. A hospital-based study in Milwaukee found positivity rates of up to 59% among Black Americans [34]. As for hospitalization [8, 34, 35], a California-based study found that they had 2.7 times higher odds of being hospitalized than their White counterparts [8]. Additionally, some studies indicated a higher mortality rate for Black Americans [4••, 36], with one study indicating mortality rates among Black Americans at 15 to 51% in varied midwestern cities [5]. Hispanic/Latino patients were also found to be more susceptible to COVID-19 [37], having the highest number of positive cases among 33 identified hotspots across the country [19] and a higher hospitalization rate (46%) in comparison to Black Americans treated for COVID-19 in a Boston-based hospital sample [7]. Additionally, Hispanic/Latino patients may have presented COVID-19 related symptoms at later stages possibly due to undocumented status and limited English ability [12, 38]. Those with lower English proficiency were shown to be particularly vulnerable as studies demonstrated that monolingual or non-English speakers were at increased odds for COVID-19–related cases [39] and hospital admissions [38, 40] but not deaths [39]. An exception to these findings of the disparate impact on racial minorities is a cohort study of 5900 patients admitted to a New York-based academic medical center in the spring of 2020 [41]. While it found that Black and Hispanic/Latino patients were more likely to test positive for COVID-19 compared to non-Hispanic whites, the survival rate from the disease did not differ by race or ethnicity after adjusting for age, sex, and related-comorbidities [41].

### HIV and Racial Health Inequities

As the COVID-19 pandemic raged throughout 2020, Black and Hispanic/Latino communities were already confronting another epidemic, particularly among young gay, bisexual and other men who have sex with men (GBMSM) [14••, 42] and heterosexual women [43, 44]. HIV rates within these racial groups have risen to a staggering rate [42, 45]. In 2018, Black Americans accounted for 42% and Hispanic/Latino Americans for 27% of new HIV diagnoses, despite comprising only 13% and 18% of the total US population, respectively [46]. In-depth qualitative interviews conducted among 50 racial minority GBMSM community and organizational leaders described the need for more awareness about biomedical HIV interventions, such as PrEP, to help reduce HIV transmission [47]. Black and Hispanic/Latina women are also at increased risk for HIV accounting for 57% and 18% at of all new HIV diagnoses among all women in the USA, respectively [46]. Previous studies showed that a 93% excess of incident HIV infections occur among Black women in comparison to the HIV incidence rate among White women [43], and that these disparities are most evident in the Northeastern and Southern USA [48]. Black community and organizational leaders indicated that gender equity, social capital, and cultural mores, such as monogamy and abstinence, were viewed as protective and could be leveraged to lower HIV risk, particularly among Black women [49]. Finally, among people living with HIV (PLWH), one study found no significant difference between White men and Hispanic/Latino Americans regardless of gender, and a small difference among Black Americans, on their engagement with HIV care, after adjusting for factors including disease stage, age, and poverty [50]. Gender may be a be factor as antiretroviral (ART) adherence and viral suppression appear to be lower among Black women, compared to Hispanic/Latina and white women [51].

## Social Determinants of Health (SDOH)

### Economic Stability: Income Inequities and Employment

#### Economic Stability and COVID-19

As the income gap in the USA continues to grow, researchers point to the parallel disparities in health, particularly among those living at and below the poverty line who have been disproportionately affected by both COVID-19 and HIV [1••, 4••, 15]. Previous studies demonstrate higher rates of COVID-19–related cases [11•, 22, 52], hospitalizations [9•, 25, 52], and deaths [12, 53] among those living in economically disadvantaged, and often more racially diverse areas. The intersection of socio-economic status and race magnifies vulnerabilities associated with poorer COVID-19 health outcomes for certain groups [11•, 12, 52]. One study of 1800 patients that found nearly 8 times higher risk of COVID-19 detection among its low-income Black patients compared to their White counterparts [22]. Another study of 2026 US counties found a 67% higher COVID-19 death rate for counties in the lowest quintile of economic privilege [53]. The arrival of COVID-19 also brought mass unemployment around the country, especially for racial groups who were overrepresented in the retail, restaurant, and hospitality industries [1••, 54]. Currently, the unemployment rate among Black versus white workers are two times higher with fewer opportunities to regain wealth after the COVID-19 pandemic [12]. These realities mirror the aftermath of the 2008 recession when White workers bounced back faster than other workers of color [12]. As such unemployment [55], along with the loss of employer issued health insurance [33, 54] led to poorer COVID-19–related health outcomes.

Individuals employed in essential industries during the pandemic, including those working in healthcare, manufacturing, waste management, construction, and warehouse sectors, were found to have higher public transportation use and lowered ability to maintain social distancing due to working outside of the home [23, 56–58]. One study estimated that approximately 16% of Hispanic/Latino and 20% of Black workers worked from home as opposed to 30% of White and 37% of Asian workers [12]. Additionally, among those who were diagnosed with COVID-19 and reported likely acquiring it at their workplace, 73% identified as Hispanic or Non-White [13]. Essential workers not only put themselves at risk for COVID-19 but by extension put at risk those living in their household, with staggering implications for multigenerational households. Using pre-pandemic data, researchers estimated 64.5% of Hispanic adults with higher risk for severe illness lived in households with at least one worker unable to work from home while Black residents were 1.6 times more likely than White residents to live with a health sector worker [59].

#### Economic Stability and HIV

Income inequality plays an important role in HIV susceptibility as well [45, 60•, 61, 62, 63, 64, 65•]. Previous studies demonstrated that residents living in high poverty US counties [45], neighborhood zip codes [66], US cities [65•], and census tracts [62, 63] had higher HIV diagnoses rates, even after controlling for other demographic factors such as marital status, education, race, and age. Interestingly, one study using an analytic sample of adolescents found that neighborhood-poverty–based differences were not significant in HIV and STI prevalence [60•]. Yet other studies have found that in certain populations, including GBMSM [61, 67] and Black women [67, 68], the risk for HIV acquisition and mortality increased for those living in higher poverty areas compared to similar populations living in lower poverty areas [67]. Researchers examining the cause behind rising HIV diagnoses and death rates among GBMSM of color, particularly in the deep US southern states, point to the higher-than-average poverty rate and limited opportunities for employment, health care access, and educational achievement [67]. Lastly, people living with HIV (PLWH) appear to be more susceptible to the negative effects of living in high poverty areas as it is associated with lower quality of life [69], unsuppressed viral load and poorer linkage to HIV care [70–72], as well as inadequate social support and health service utilization [73, 74]. Conversely, employment stability has been shown to improve or maintain HIV care engagement among PLWH [75, 76].

## Neighborhood and Built Environment: Housing and Population Density

### Neighborhood and Built Environment and COVID-19

COVID-19 brought to light the importance of the built environment in mitigating infectious disease [10•, 11•, 77]. Previous studies demonstrated that higher household density, defined as residences with more than one person per room, multigenerational households, or overcrowded homes increased the transmission risk of COVID-19 among Hispanic/Latino Americans [12, 23, 39, 52, 78]. While both Black and Hispanic/Latino residents were found to have a higher likelihood of receiving a COVID-19 diagnosis if they lived in more densely populated communities [79], a spatial analysis of COVID-19 hotspots indicated that housing density had a stronger association with hotspots than population density [23]. Additionally, low-income families situated in more coveted and gentrified urban areas with rising property and rental rates find themselves being led to crowd in or “double up” within tightly spaced homes [58, 80]. Communities of color are particularly vulnerable to housing displacement, as Black women often face eviction after a job loss [81] while up to 70% of African and Southeast Asian refugee families living in the Southeast USA reported fear of income loss during the pandemic [82]. Hispanic/Latino adults were also found to have higher prevalence of psychosocial stress compared to other racial groups, due to concerns of not having enough food or stable housing during the COVID-19 pandemic [83]. The lack of housing stability was also found to be associated with COVID-19, as 24% of COVID-related hospital admissions were among those experiencing homelessness [7]. With lower access to hygiene facilities, living with others in congregate settings, and higher rates of chronic physical and mental illness, those experiencing homelessness were shown to be at higher risk for COVID-19 acquisition [84].

### Neighborhood and Built Environment and HIV

While high housing density has not shown to be a significant factor in mitigating HIV transmission, housing stability has been found to help improve HIV care engagement among PLWH [72, 77, 85–87]. Among PLWH who received housing support, 86% indicated viral suppression success [72]. Stable housing offers PLWH increased access to social support and health care services as well as lowers their risk of assault and coercion [77, 85]. Additionally, in a study that examined a 2018 outbreak of HIV among people who inject drugs (PWID), increased homelessness was cited as one of the likely factors behind the outbreak [86]. Demographic density also appears to reduce HIV acquisition, particularly for men who have sex with men (MSM). MSM living in areas with a higher percentage of lesbian, gay, bisexual and transgender (LGBT) persons were found to have a lower HIV diagnosis rate compared to MSM living in less LGBT-populous communities, suggesting that living in more LGBT-friendly communities can be protective against HIV transmission [61].

## Social and Community Support: Stigma, Discrimination, Norms and Social Networks

### Social and Community Support and COVID-19

Infectious diseases have long been viewed as “foreign” entities brought to the USA by non-citizens who are believed to be “different” from the general population [14••, 88]. In the 1980s, initially Black Haitian foreigners then eventually young, predominantly white gay men, were stigmatized owing to a then-emerging virus now known as HIV and AIDS [88, 89], while in 2020, the former US president aimed to characterize COVID-19 as a “Chinese virus,” which lead to a wave of xenophobic acts across the country [89, 90, 91]. Specific populations who are viewed to be “carriers” of particular infectious diseases are subjected to discriminatory behavior by the general public, which can lead to isolation and overall poorer quality of life. In a study conducted at the start of the COVID-19 pandemic, individuals who reported experiencing COVID-19–related discrimination, including being treated with less courtesy and respect or feeling that others are afraid of them, went up among Black and Asian US residents, from 9 to 15% and 11 to 17%, respectively [92•]. Another study reported that Asian Americans’ anxiety, depressive symptoms, and sleep difficulties rose 40% and experienced discrimination increased 30% since the beginning of the COVID-19 pandemic, with social support shown to buffer the negative effects of discrimination in this population [93]. A robust social network has also proven to ameliorate stigmatization and negative outcomes of COVID-19 among Black Americans, despite a study finding that collective engagement in predominately Black residential areas was associated with a higher COVID-19 diagnosis rate [94]. Within the community context, studies indicate that building a network of trusted community sources, largely via word-of-mouth, can help dispel the uncertainty and myths surrounding COVID-19 prevention and treatment among Black Americans [95, 96]. The role of trusted social networks to provide accurate information may be particularly important as Black communities have often been subjected to various unsubstantiated characterizations of being immune to certain diseases and infectious agents due to their genetic make-up [97].

### Social and Community Support and HIV

Among these enduring myths is that Black Americans are not susceptible to HIV [97]. Despite the alarming rate of HIV among Black women and Black GBMSM in particular, previous studies noted that the deep religiosity, conservatism, and homophobia within the Black community have allowed this myth to thrive, thus generating intense stigma surrounding HIV [65•, 67, 73]. In fact, stigma related to HIV and same sex sexual behaviors is found to be high among Black Americans compared to other racial groups [65•, 67, 98•], which has impacted HIV prevention and care engagement efforts such as the willingness to test and treat HIV [67, 89]. Hispanic/Latino and Indigenous communities have also seen a rise in HIV and attribute the rapid transmission among its members to HIV stigma and discrimination [99, 100]. Indeed, stigma has been associated with perceived loss of status, ostracization and devaluation [89], and PLWH are acutely aware of these negative effects [73, 101, 102]. PLWH have reported reduced life chances in the form of unemployment or under-employment [67, 73], poor to fair self-rated health [103], and unfair treatment by health and social service professionals [47, 102].

Conversely, social support has helped mitigate HIV stigma and discrimination among PLWH [69, 73, 104]. In one study, young gay and bisexual men (GBM) and transgender women of color reported that increased interaction with other members of the LGBT community via group and community activities, such as local Pride festivals, and building informal support networks composed of allies and partners are effective in reducing stigma and can help improve HIV prevention and care engagement for this population [47]. Among Black PLWH, instrumental support from family members was shown to be beneficial in filling the gaps left by governmental social service agencies as well as in reducing stigma, as some participants stated that their interaction with family members was surprisingly positive when their HIV status was discussed [73, 105]. Yet greater societal and institutional support may also have an effect on population-level HIV incidence and treatment engagement [65•, 67, 101]. Laws permitting same-sex marriage were associated with lower HIV incidence among MSM [65•], while the mortality rate among PLWH was found to be lower in US states with anti-discrimination laws [101], compared to other states without similar regulations.

## Healthcare Access and Quality

### Healthcare Access and Quality and COVID-19

Long before COVID-19 became a global pandemic, the documented discrimination, harm and abuse of authority within the US medical, health, and social services institutions against low-income racial minorities have generated a deep distrust among communities of color [1••, 12, 89]. The generational distrust of such institutions has weakened its ability to communicate appropriate and relevant COVID-19 information to racial minority communities disproportionately affected by the disease [95, 106], which has led to lower testing [96] and potentially later stage presentation of COVID-19 at the hospital [12, 107]. Early in the COVID-19 pandemic, most of the testing centers were situated in largely White and more affluent neighborhoods [10•, 54], which only exacerbated the multiple healthcare-related barriers facing minority communities with increased risk for COVID-19 exposure [33, 108]. Retaining comprehensive health insurance is a significant factor, particularly for individuals who are at risk of job loss, as they may have difficulty in seeking care for COVID-related symptoms without employer-issued health insurance [12, 53, 82]. In a review of medical records for patients admitted during the early months of the pandemic, Hispanic/Latino patients were shown to have a higher percentage of self-pay health insurance as well as having higher odds of testing positive for COVID-19 compared to other racial groups [109]. Finally, while telehealth services, defined as the provision of health services via telecommunication technologies, have been shown to improve health access for disparate and marginalized populations [107], new evidence indicates that low-income rural, racial minority, and elderly communities may be excluded from this novel service due to patients’ lower digital technology literacy and insufficient resources of underfunded clinics and hospitals [96, 110].

### Healthcare Access and Quality and HIV

Mistrust of healthcare and social services institutions is particularly evident among PLWH, with a focus on provider-level issues and interactions [47, 111]. Previous studies demonstrated that HIV stigma perpetuated by health care and social services providers is a source of concern among racial minority PLWH [102] and is associated with low linkages to care after an HIV diagnosis [112]. Additionally, community clinics facing fiscal constraints, especially in low-income and racially segregated neighborhoods, have left PLWH with restricted choices for HIV services due to reduced clinic hours and minimum staff availability [98•]. Low-income PLWH must also contend with the limited availability of health services for public health insurance recipients, which has been shown to influence the availability and quality of HIV care in this population [2••, 68, 101]. For example, previous studies indicated that optimal HIV viral suppression is associated with the use of private versus public health insurance and clinics [51], while the mortality risk among PLWH was lower for those living in states with greater health care coverage [68, 101]. Yet not all areas of the country are afforded equal healthcare availability as evidenced in the southern US states, wherein physician visits and pharmacy benefits are heavily regulated due to restrictive Medicaid provisions [67]. These southern US states must rely on other federal programs, such as the Ryan White Care program, to provide sufficient HIV care for PLWH [67].

## Educational Access and Quality

Multiple studies demonstrate that educational achievement, along with other social determinants such as employment, healthcare availability, and poverty, can impact COVID-19–related transmission [23, 28], hospital admissions [26], and deaths [30, 53, 55]. Areas with a higher percentage of residents without a high school diploma are associated with increased COVID-19–related mortality rates [28, 30, 53], while hospital admissions during the early months of the COVID-19 pandemic was shown to be correlated with neighborhoods with the least proportion of residents with a college degree [26]. Similarly, HIV care engagement, including optimal viral suppression, appears to be lower among PLWH who did not complete a high school education [87, 112]. However, educational achievement was not found to be correlated with HIV transmission [45, 113], despite the expectation that higher levels of education is equated with better health outcomes [100]. In studies examining the rates of HIV diagnoses within the state of Mississippi [113] and in a sample of 1560 counties across the USA [45], having less than a high school education was either negatively associated with [113] or not indicated [45] for higher HIV diagnoses rates.

## Conclusion

This literature review clearly demonstrates the socially determined health inequities among communities of color deeply affected by COVID-19 and HIV. Such inequities are a result of racialized realities that are impacted by economic instability, the built environment, limited healthcare access and quality as well as lowered educational opportunities. Additionally, stigma and discrimination have shown to exacerbate these health inequities but can be mitigated by social and community-level supports. The structural and systematic factors that drive HIV and COVID-19 transmission highlight the importance of not solely focusing on biomedical interventions as solutions to ending HIV and COVID-19, but rather call for building a more just public health and social service safety net that meets the needs of people at the intersection of multiple vulnerabilities.

## Data Availability

N/A.

## Code availability

N/A.
